# Beyond one-size-fits-all: mapping information-seeking and decision-making pathways in cancer care

**DOI:** 10.1007/s00520-026-11250-4

**Published:** 2026-09-22

**Authors:** Neta Shanwetter Levit, Mor Saban

**Affiliations:** School of Health Professions, Gray Faculty of Medical and Health Sciences, Tel Aviv University, P.O. Box 39040, 69978 Tel Aviv, Israel

**Keywords:** Patient-centered care, Shared decision-making, Oncology, Decision support, Cancer-care navigation

## Abstract

**Purpose:**

To identify distinct archetypes among adult cancer patients based on information-seeking patterns from symptom onset to treatment initiation, including the use of digital and emerging technologies such as generative AI, and to characterize the decision-making dilemmas associated with these pathways.

**Methods:**

We conducted a cross-sectional study in Israel, between February 2025 and December 2025, among 205 adult cancer patients to examine patterns of information seeking and decision-making during the period from symptom onset to treatment initiation. Participants completed a structured questionnaire assessing reliance on multiple information sources—including medical professionals, family members, digital resources, and generative AI-based tools—before and after diagnosis, along with sociodemographic and clinical characteristics. Patient archetypes were identified using cluster analysis, and decision-making dilemmas were explored using two methods (investigator-led constant comparative analysis and LLM) for open-ended responses.

**Results:**

Four distinct information-seeking archetypes were identified: Digital Natives (27.6%), Family-Centered (38.8%), Balanced Traditional (25.0%), and Medical Professional-Focused (8.6%). Archetypes differed significantly by age, education, and the strongest differentiating factor (*p* = 0.005)—religiosity. Across archetypes, information seeking intensified after diagnosis, relying mostly on family members, internet sources, and additional medical professionals, whereas reliance on generative AI-based tools remained consistently low. Among respondents to the open-ended question, 86% reported significant decision-making dilemmas, most commonly related to treatment selection and choice of healthcare provider or facility.

**Conclusion:**

The marked heterogeneity observed in patients’ information-seeking and decision-making pathways highlights the inadequacy of one-size-fits-all approaches in early cancer care. Implementing tailored, culturally responsive decision support may better align care with patients’ needs during this critical phase.

**Supplementary Information:**

The online version contains supplementary material available at https://doi.org/10.1007/s00520-026-11250-4.

## Introduction

The landscape of cancer treatment and care has shifted in the last decade from a clinician-centered, paternalistic model toward patient-centered care and shared decision-making, in which patients are expected to take an active role in navigating their treatment and care pathways [1, 2]. In oncology, this shift is particularly relevant, as patients are often required to make complex decisions under conditions of uncertainty, emotional distress, and time pressure [3, 4]. As a result, decision-making in cancer care extends beyond discrete clinical encounters and unfolds across a broader trajectory that includes symptom appraisal, diagnostic processes, and treatment initiation [4].

Growing attention has therefore been directed to understanding how patients seek, interpret, and integrate information when making health-related decisions [5, 6]. Research suggests that patients rarely rely on a single source of information; instead, decision-making is shaped by interactions among multiple medical, social, and contextual influences [7, 8]. Although physicians remain a central source of authority and guidance, family members, partners, peers, and community networks often play substantial roles in framing options, providing emotional support, and influencing preferences [9, 10]. These interpersonal dynamics are particularly salient in serious illnesses such as cancer, where decisions frequently carry profound personal, social, and existential implications [11, 12].

Beyond interpersonal influences, the informational environment surrounding cancer care has expanded considerably with the proliferation of digital health technologies [13, 14]. Patients are increasingly engaging in online health-information seeking—consulting medical websites and databases, participating in patient communities, and drawing on social media and peer-generated content to supplement clinical advice [15]. Prior studies have shown that such engagement may enhance patients’ sense of control, preparedness, and involvement in care [14, 16]. At the same time, exposure to multiple and sometimes conflicting sources of information may increase cognitive burden, exacerbate decision uncertainty, and complicate efforts to reconcile professional recommendations with personal values and social expectations [17, 18].

Within this evolving digital information landscape, tools based on artificial intelligence (AI), including generative AI (GenAI), have recently emerged as potential sources of health information and decision support [14, 19]. Existing literature on AI in healthcare has largely focused on system performance and accuracy, ethical considerations, and comparisons between AI-generated and clinician-provided information [20, 21]. Some studies have explored the potential of AI-driven tools to support patient education, improve accessibility to medical information, or facilitate shared decision-making [22]. However, empirical evidence regarding how patients actually engage with AI-based tools during real-world cancer care remains limited. In particular, little is known about how such tools are integrated—if at all—into patients’ broader information environments alongside physicians, family members, online resources, and peer networks [23].

Despite increasing recognition of the complexity of patient decision-making, current research lacks comprehensive models that capture the full informational ecology in which cancer-related decisions are made [24, 25]. Many existing frameworks focus on isolated decision points or emphasize individual factors, such as health literacy or decisional conflict, without accounting for the dynamic interplay among multiple sources of influence across the care journey [26]. Similarly, available instruments often assess patient satisfaction or experience but do not explicitly map how patients prioritize and combine different informational inputs when navigating key decisions [27, 28].

To address these gaps, the present study adopts a patient-centered perspective to examine how individuals perceive and prioritize diverse influential sources when making cancer-related decisions from symptom onset to treatment initiation. By mapping information-seeking patterns and identifying common decision-making dilemmas, this study aims to move beyond one-size-fits-all models of patient engagement and contribute to a more nuanced understanding of decision-making within contemporary cancer-care environments.

## Methods

### Study design

**Fig. 1:**
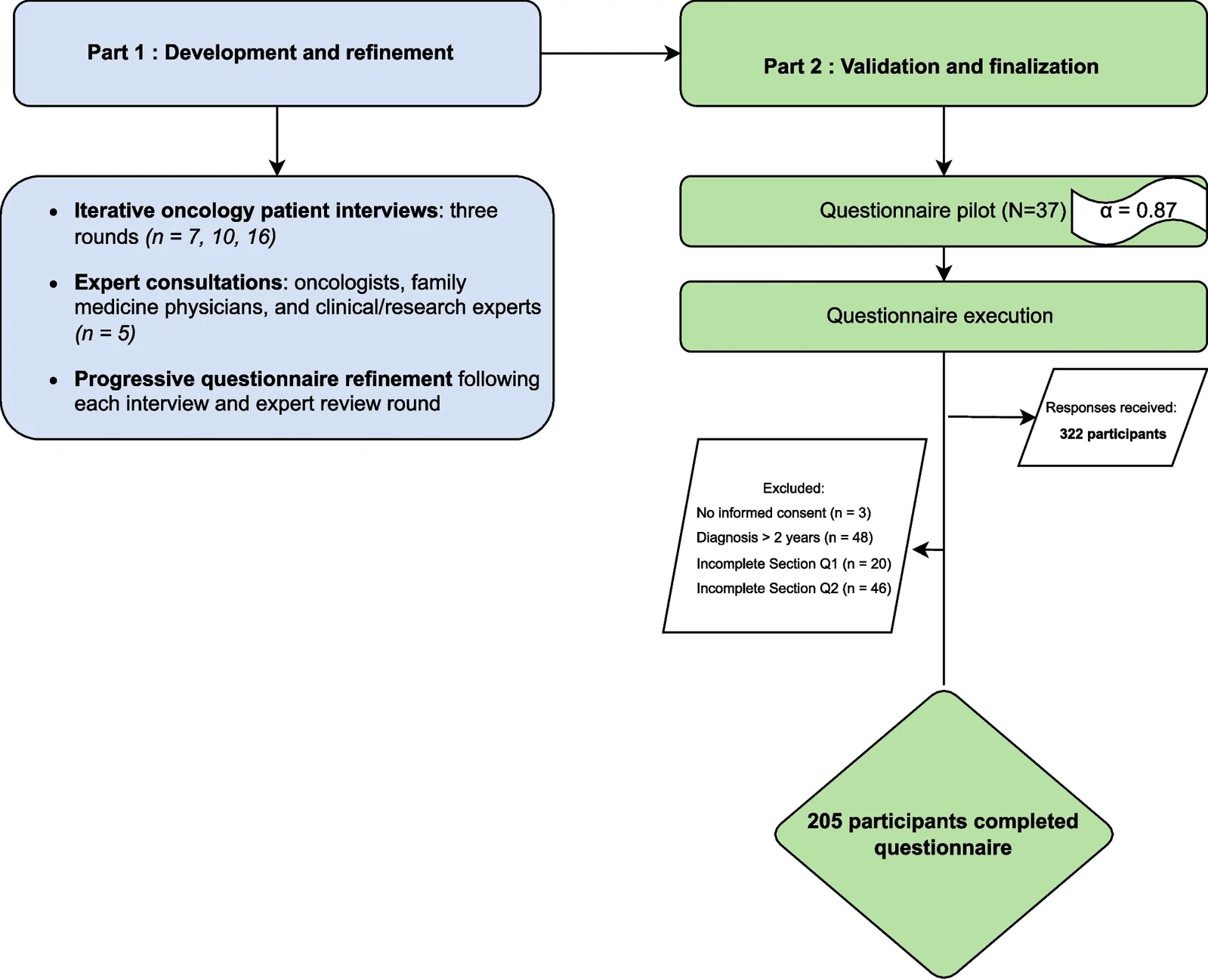
Study design and questionnaire-development process

This study employed a patient-centered mixed-methods approach with preparatory instrument development followed by cross-sectional empirical investigation. The study was conducted in Israel between February 2025 and December 2025. Qualitative interviews and pilot testing were conducted first to develop and refine a questionnaire capturing oncology patients’ decision-making processes. The finalized instrument was then applied to examine patterns of information seeking and decision influences across the cancer-care journey (Fig. 1).

### Participants and recruitment

Adult cancer patients were recruited through patient-led online communities, primarily cancer-related Facebook support groups. A snowball sampling strategy was used, whereby participants were encouraged to share the questionnaire with other eligible patients, enabling recruitment beyond a single group and facilitating participation among individuals with diverse cancer experiences. Participants were eligible for inclusion if they were 18 years of age or older, had received a cancer diagnosis within the previous 2 years, and provided informed consent to participate; and excluded if they had received their first cancer diagnosis more than 2 years prior to questionnaire completion or did not complete the consent process. Participation was voluntary and anonymous. To minimize duplicate responses, IP addresses were used solely to identify and remove multiple questionnaire submissions originating from the same source prior to analysis. No personally identifiable information was collected or stored. The study was approved by the Tel Aviv University Ethical Review Board (approval number 0008350-4) and conducted in accordance with the Declaration of Helsinki; all participants provided informed consent prior to questionnaire completion.

### Questionnaire development and refinement (preparatory phase)

The Patient Decision Agents Questionnaire (PDAQ) was developed through a systematic, patient-centered process that combined iterative qualitative interviews, expert consultations, and pilot testing to ensure relevance, clarity, and reliability.

#### Iterative patient interviews

Three rounds of semi-structured interviews were conducted with adult oncology patients (*n* = 7, 10, and 16 in rounds 1, 2, and 3, respectively). Interviews explored patients’ experiences from symptom onset through treatment initiation, with particular emphasis on key decision points along the care journey, perceived decision agents (including medical professionals, social networks, digital sources, and AI), information-seeking behaviors, and decision dilemmas.

Interview transcripts from all three rounds were analyzed using thematic content analysis. The first and second authors independently coded the data using an iterative approach, with discrepancies resolved through discussion until consensus was achieved. Identified themes informed questionnaire-item generation, wording, and structural organization. The semi-structured interview guide is provided in Online Resource 1.

##### Expert consultations

In parallel with patient interviews, expert consultations were conducted with senior oncologists, family medicine physicians, and clinical and research experts (*n* = 5). Experts reviewed questionnaire items for clinical relevance, conceptual clarity, and alignment with patient-centered care and shared decision-making frameworks. Feedback from experts was synthesized and integrated alongside patient input to guide successive revisions of the questionnaire.

##### Pilot testing and questionnaire finalization

The refined questionnaire was pilot-tested with 37 oncology patients representing a range of cancer types—breast, melanoma, thyroid, ovarian, and lymphoma. The pilot phase assessed feasibility, clarity, and internal consistency. Characteristics of the pilot sample are presented in Online Resource 2.

Internal consistency of multi-item scales was evaluated using Cronbach’s alpha, yielding a coefficient of 0.87, indicating good reliability. Item–total correlations identified items requiring revision or removal. Participant feedback regarding clarity and comprehension informed final wording and structural adjustments prior to large-scale deployment. The full reliability analysis is provided in Online Resource 3.

The final questionnaire (Online Resource 4) comprised three sections:

1. Chronological mapping of the patient’s journey from symptom onset to treatment initiation
2. Patient-rated influence of decision agents, assessed using a five-point Likert scale across two time periods (symptom onset to diagnosis; diagnosis to treatment initiation)
3. Decision dilemmas and care-management responsibilities

### Questionnaire administration and data collection

The finalized questionnaire was administered online to the full study sample (*n* = 205). Participants completed the questionnaire independently, reporting on their information sources, decision-making experiences, and sociodemographic and clinical characteristics. Responses were collected anonymously and stored securely for analysis. Household income was self-reported relative to the average national monthly wage in Israel, on a five-point scale ranging from far below average to far above average.

### Data analysis

Descriptive statistics were used to characterize the final study sample of 205 participants with complete questionnaire data. Changes in reliance on information sources before and after diagnosis were examined using paired *t*-tests across 11 information sources, with Bonferroni correction applied to account for multiple comparisons. Effect sizes were calculated using Cohen’s d.

To identify distinct information-seeking patterns, *K*-means cluster analysis (*k* = 4) was conducted using 22 variables representing reliance on 11 information sources, before and after diagnosis. Cluster analyses were conducted using complete case analyses and therefore included only participants with complete data across all 22 information-source variables (*n* = 152). All other analyses were conducted using the full available sample (*n* = 205). The optimal number of clusters was based on convergence across multiple indices, including the silhouette coefficient, Calinski–Harabasz index, and gap statistic. Differences in demographic and clinical characteristics across identified clusters were examined using chi-square tests for categorical variables and one-way analysis of variance with Tukey post hoc comparisons for continuous variables.

Responses to open-ended questions regarding decision-making dilemmas were analyzed using directed qualitative content analysis. The first author conducted the primary coding. The large language model (LLM) ChatGPT 5.2 OpenAI was used as an independent second reviewer to support code validation, following a previously published and validated methodology [4]. Inter-rater reliability between the human and LLM-assisted coding was assessed using percentage agreement. Cohen’s kappa (0.85) demonstrated high agreement (89%). Discrepancies were resolved through discussion and review by the second author, followed by consensus coding. Frequencies were calculated for each dilemma category.

Statistical significance was set at *α* = 0.05 (two-tailed). For paired comparisons, the Bonferroni-adjusted significance threshold was *α* = 0.0045 (0.05/11). All analyses were conducted using R version 4.2.1, SPSS version 28.

## Results

### Participant characteristics

The final analytical sample included 205 participants. Their characteristics are reported in Table 1. The sample was predominantly female. Educational attainment was high, and most participants were married. Religiosity distribution was mostly secular, and private health insurance was reported by over two-thirds of the participants.

**Table 1:** Patients’ sociodemographic and clinical characteristics

Characteristic	Value
Gender, ***n*** (%)	
Male Female Missing values	35 (17.1) 168 (82.0) 2 (1.0)
**Age range in years (mean, SD)**	22–79 (57.5, 10.8)
**Family status, *n* (%)**	
Married In a relationship Single Widowed Divorced/separated Missing values	139 (67.8) 21 (10.2) 21 (10.2) 3 (1.5) 19 (9.3) 2 (1.0)
**Religiosity,** ***n*** (%)	
Secular Traditional Religious Ultra-Orthodox Other Missing values	144 (70.2) 31 (15.1) 24 (11.7) 2 (1.0) 2 (1.0) 2 (1.0)
**Years of education range (mean, SD)**	5–28 (16.6, 3.4)
**Self-reported average income,** ***n*** (%)	
Far below average Below average Around average Above average Far above average Missing values	24 (11.7) 28 (13.7) 51 (24.9) 75 (36.6) 22 (10.7) 5 (2.4)
**Employment status,** ***n*** (%)	
Employed Unemployed Retiree/pensioner Self-employed On medical leave/work disability Missing values	109 (53.2) 7 (3.4) 42 (20.5) 34 (16.6) 10 (4.9) 3 (1.5)
**Additional private insurance,** ***n*** (%)	
Yes No Missing values	142 (69.3) 58 (28.3) 5 (2.4)
**Cancer staging in diagnosis,** ***n*** (%)	
Initial Advanced Missing values	120 (58.5) 76 (37.1) 9 (4.4)
**Cancer types,** ***n*** (%)	
Breast cancer Genitourinary cancers (prostate, bladder, testicular) Gynecological cancers (ovarian, uterine, cervical) Gastrointestinal cancers (colon, rectal, stomach, pancreatic, liver) Thoracic cancers (lung) Hematologic malignancies (lymphoma, leukemia) Skin cancers (melanoma, SCC) Neuroendocrine tumors Other/unclear	108 (52.7) 28 (13.7) 10 (4.9) 10 (4.9) 12 (5.9) 6 (2.9) 4 (2.0) 2 (1.0) 25 (12.2)

Cancer staging at diagnosis and cancer type are presented in Table 1, with breast cancer being the most common diagnosis. To improve interpretability and avoid over-fragmentation of rare malignancies, remaining diagnoses were grouped into broader categories, including other solid tumors (*n* = 74, 36.1%), hematologic malignancies, and missing or unclear diagnoses. Cancer type was not treated as a primary analytical driver because information-seeking behaviors were assessed across the early-care trajectory, including the pre-diagnostic phase, during which many participants had not yet received a definitive cancer diagnosis when engaging with information sources.

### Changes in information-source utilization before and after diagnosis

#### Pre-diagnosis information sources (from symptom till diagnosis)

The most utilized sources were internet search (M, SD: 3.10, 1.38), family/spouse (2.89, 1.42), and social media (2.52, 1.45). Use of medical databases (2.39, 1.41), friends/community (2.30, 1.36), and patient organizations (1.81, 1.23) was moderate. Least utilized were AI tools (1.67, 1.12), alternative medicine (1.49, 0.98), and religious/spiritual guidance (1.30, 0.76).

### Post-diagnosis information sources (diagnosis-to-treatment initiation phase)

Significant increases occurred for family/spouse (3.27, 1.38, *p* < 0.001), internet search (3.20, 1.36, *p* = 0.030(, other medical providers (2.95, 1.48, *p* < 0.001), social media (2.75, 1.52, *p* < 0.001), and family physician (2.19, 1.35, *p* < 0.001). No significant changes were observed for medical databases (*p* = 0.89), patient organizations (*p* = 0.12), AI tools (*p* = 0.18), alternative medicine (*p* = 0.34), or religious/spiritual guidance (*p* = 0.45).

**Fig. 2:**
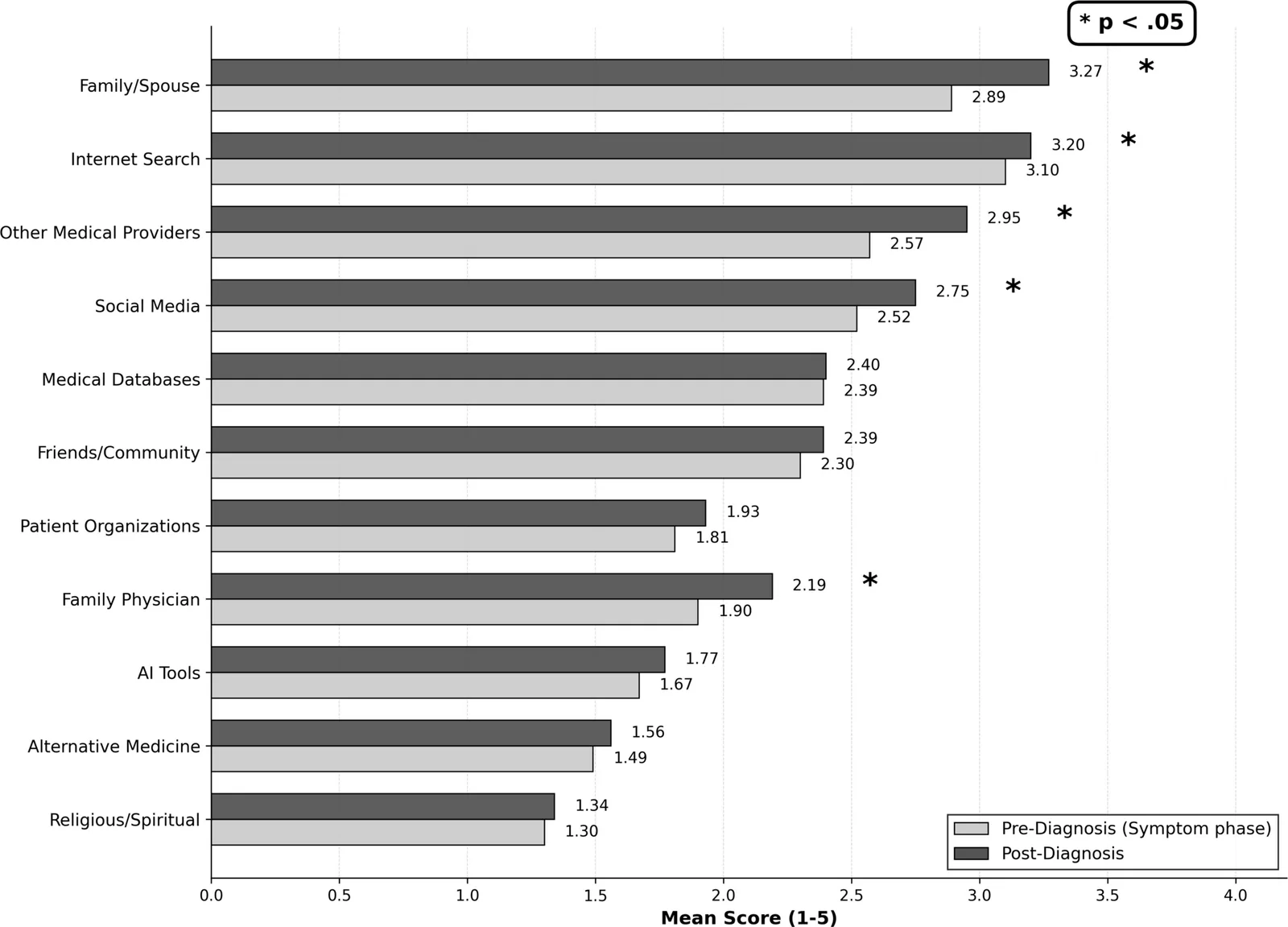
Pre- and post-diagnosis differences in patient-reported information sources

AI tools remained the least utilized digital resource throughout the patient journey, even among younger participants (Fig. 2).

### Patient archetypes based on information-seeking patterns

**Fig. 3:**
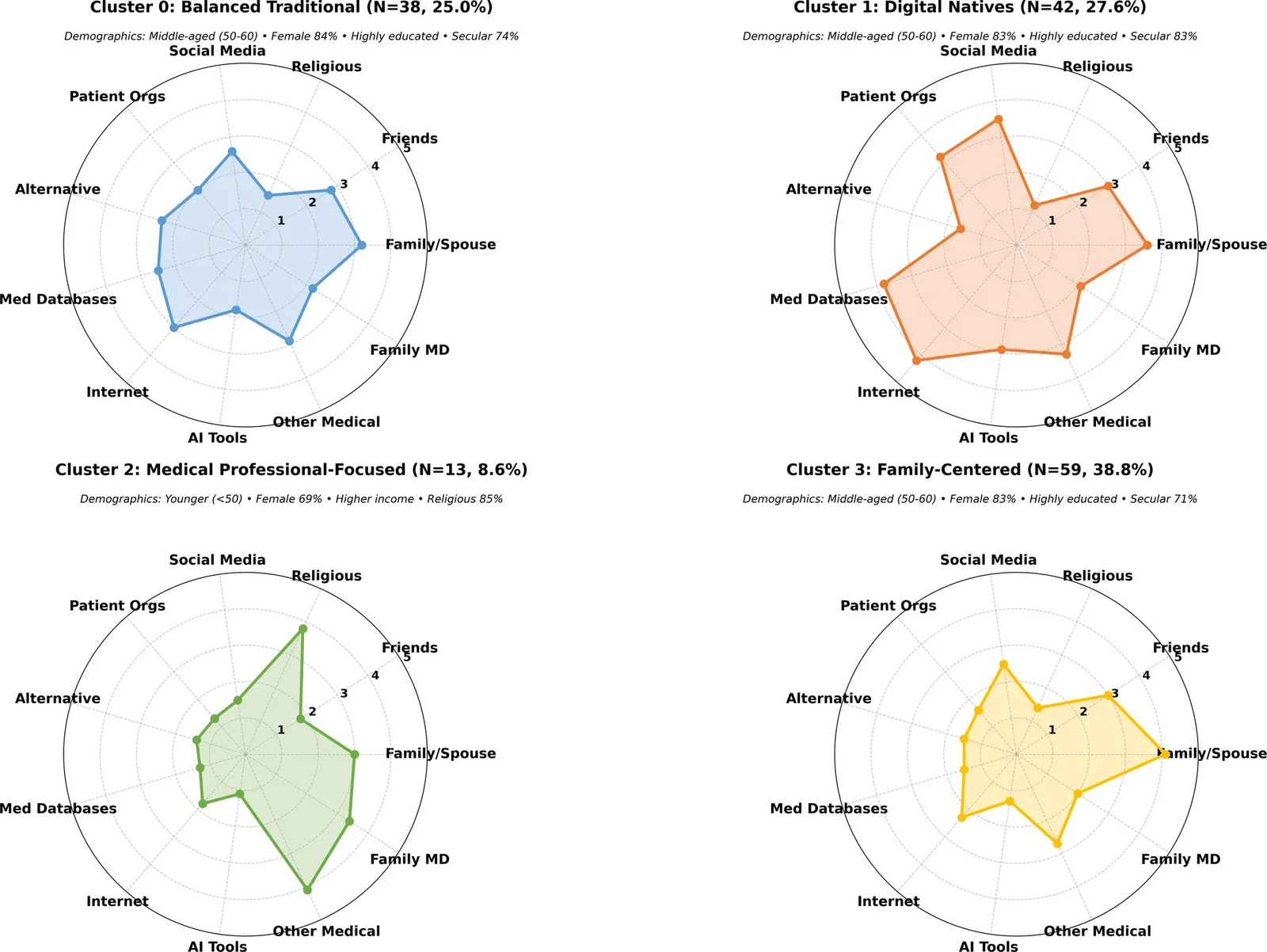
Patient archetypes based on information-source utilization patterns

K-means cluster analysis identified four distinct patient archetypes based on information source-utilization patterns. Analysis was performed on participants with complete data on all 22 information-source variables (11 sources × 2 time periods). Cluster validity indices supported the four-cluster solution: silhouette coefficient = 0.42, Calinski–Harabasz index = 187.3, gap statistic peaked at *k* = 4 (Fig. 3).

### Cluster 0: Balanced Traditional

This archetype was characterized by moderate, steady engagement across multiple information sources without extreme reliance on any single channel. These patients demonstrated pragmatic flexibility, consulting internet sources moderately (*M* = 3.0 post-diagnosis), maintaining balanced connections with both family (3.2) and medical professionals (3.0), and showing openness to alternative approaches (2.4). This middle-aged group (*M* = 54.9 years) was predominantly female (84.2%), highly educated (16.6 years), and secular (73.7%), with mixed employment status and half reporting above-average income.

### Cluster 1: Digital Natives

This archetype was characterized by the highest overall level of information seeking, driven primarily by digital sources. These patients actively curated information through internet search engines (*M* = 4.2 post-diagnosis), medical databases (3.8), and social media health communities (3.5), while also being the most frequent AI-tool users (2.9) despite overall low AI adoption. They maintained strong connections with patient organizations (3.2), suggesting a comprehensive approach to information gathering. This middle-aged cohort (*M* = 51.7 years), predominantly female (83.3%), highly educated (16.6 years), and secular (83.3%), demonstrated higher employment rates (52.4%) and lower marriage rates (61.9%) than other groups.

### Cluster 2: Medical Professional-Focused

This was the smallest group; these patients showed very high reliance on physician authority alongside minimal engagement with digital information sources. They reported the strongest reliance on their primary oncologist (*M* = 4.5—the highest score observed across all sources and archetypes) and other medical specialists (4.1), with limited use of internet sources (1.8), medical databases (1.3), and AI tools (1.1)—the lowest values across groups. This cohort was the youngest on average (*M* = 49.0 years), had lower female representation (69.2%), and the highest marriage rates (84.6%). A large majority (84.6%) identified as traditional, religious, or ultra-Orthodox, compared to the predominantly secular identification in other clusters (71–83%). This group also reported the highest income levels (53.8% above average) and employment rates (53.8%).

### Cluster 3: Family-Centered

This was the largest group; these patients relied predominantly on interpersonal relationships for information and decision support. They showed the strongest family/spouse reliance across all archetypes (*M* = 4.1 post-diagnosis), supplemented by moderate friend and community connections (3.0), while demonstrating minimal engagement with digital channels—internet (2.3), medical databases (1.5), and AI tools (1.3). This middle-aged (*M* = 53.4 years), predominantly female (83.1%), highly educated (16.2 years), and secular (71.2%) cohort, had the highest employment rates (61.0%).

### Archetype differentiation

Statistical comparisons revealed significant differences across archetypes in: religiosity (*χ*^2^(9) = 21.5, *p* = 0.005, Cramér’s *V* = 0.25)—the strongest differentiator; age (*F*(3,148) = 8.3, *p* < 0.001, *η*^2^ = 0.14, with Digital Natives being significantly younger); education (*χ*^2^(6) = 16.2, *p* = 0.012, Cramér’s *V* = 0.23); health literacy (*F*(3,148) = 12.1, *p* < 0.001, *η*^2^ = 0.20); and self-management confidence (*F*(3,148) = 9.7, *p* < 0.001, *η*^2^ = 0.16). No significant differences were found for gender, marital status, income, or cancer type.

### Decision-making challenges and dilemmas

**Fig. 4:**
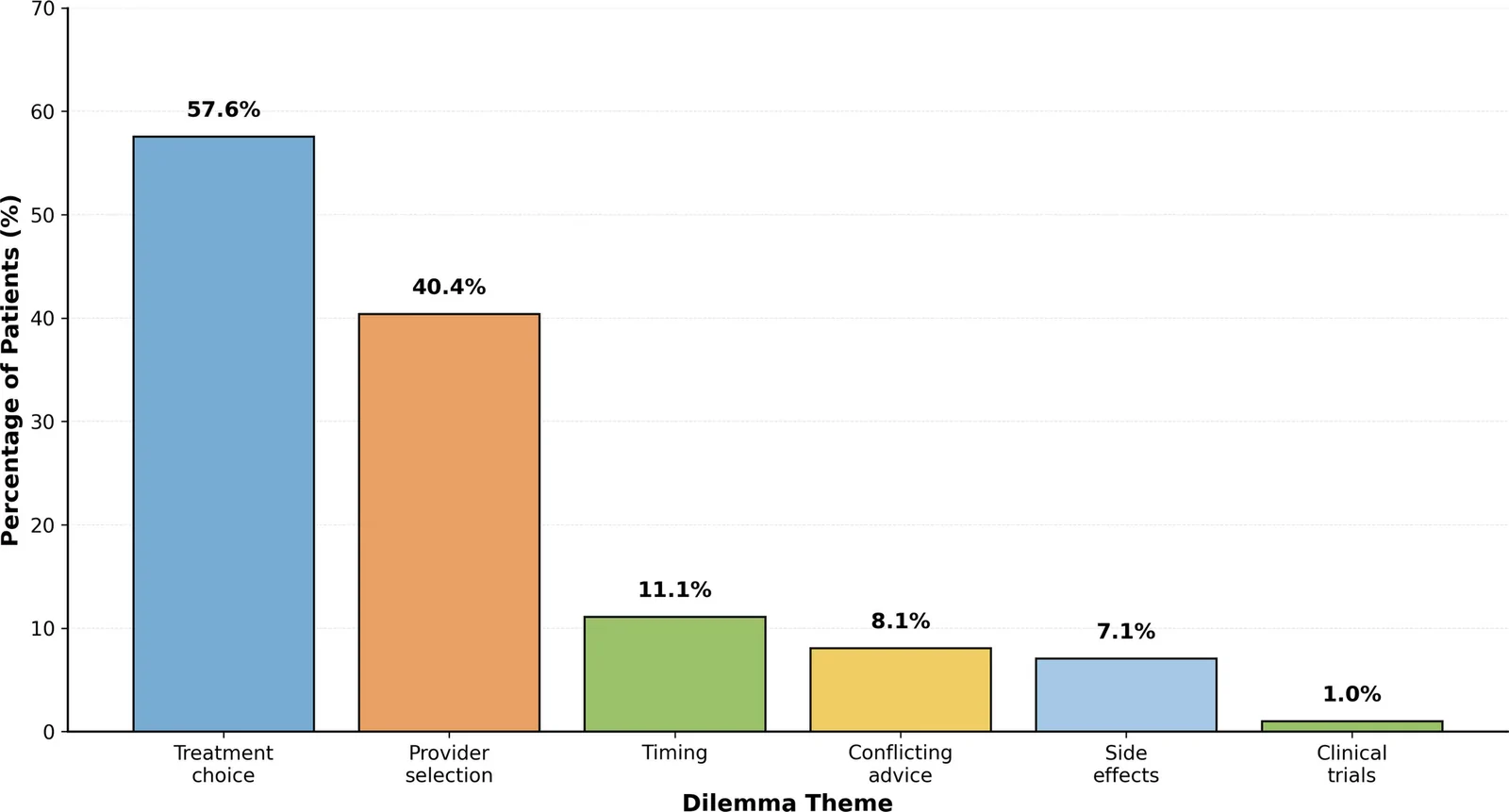
Patient-reported decision-making dilemmas and thematic distribution

Of the 205 participants, 115 (56.1%) responded to open-ended dilemma questions; 99 (86.1%) reported significant dilemmas and 16 (13.9%) reported none. Content analysis revealed two dominant themes (Fig. 4): Treatment Choice (*n* = 57)—selecting among chemotherapy, biological therapy, radiation, clinical trials, or surgical versus non-surgical approaches; and Provider/Facility Selection (40)—choosing oncologists and hospitals by balancing expertise, accessibility, wait times, and resources. Minor themes included Timing/Urgency (11), Conflicting Medical Opinions (8), Side Effects/Quality of Life (7), and Clinical Trial Participation (1).

## Discussion

This study provides a patient-centered examination of how individuals navigate cancer-related decision-making across their care journey. We found that decision-making from symptom onset to treatment initiation is shaped by a constellation of medical, social, and informational influences, rather than occurring solely within discrete clinical encounters. Patients described themselves as active participants in managing their care, integrating input from clinicians, family members, and diverse information sources while confronting recurring decision challenges.

### Information-seeking archetypes

The identification of four distinct information-seeking archetypes extends theoretical understanding of patient heterogeneity in health decision-making [29–31]. Rather than viewing information seeking as a continuum from low to high engagement, these findings suggest qualitatively different approaches reflecting distinct underlying preferences, capabilities, and values. The Digital Natives archetype exemplifies how younger, highly educated patients leverage digital tools for active information curation and verification, consistent with self-determination theory’s emphasis on autonomy and competence [31, 32]. In contrast, the Family-Centered archetype reflects a collectivist approach where decision-making authority and information processing are distributed across family networks rather than concentrated in the individual patient [11]. The Medical Professional-Focused archetype is strongly associated with religiosity, suggesting that for some patients, trust in physician authority represents active alignment with deeply held cultural and spiritual values about medical decision-making, rather than passive abdication [33]. These findings challenge deficit-based models that view lower information seeking as “disengagement,” instead recognizing diverse pathways to meaningful participation in care [34].

The emergence of religiosity as the strongest archetype differentiator provides important insights into how cultural frameworks shape health behavior. Previous research has documented religious influence on specific medical decisions—such as end-of-life care or genetic testing, but less on how religiosity fundamentally structures the decision-making process itself [35–37]. The high concentration of religious/ultra-Orthodox patients in the Medical Professional-Focused archetype suggests that religious worldviews foster distinct epistemological assumptions about medical knowledge and authority. For religious patients, physician recommendations may carry weight not only as clinical expertise but also as alignment with religious values of respecting authority and divine providence in health outcomes [38]. This finding has implications beyond religious communities, highlighting how cultural identity—whether defined by religion, ethnicity, or other dimensions—creates coherent frameworks for navigating medical uncertainty that may differ substantially from mainstream biomedical assumptions about patient autonomy and information seeking [5, 39, 40].

### Changes in information-source utilization

The evolution of information-source utilization from pre- to post-diagnosis sheds light on the adaptive nature of patient decision-making. Significant increases in family consultation, internet searching, and engagement with additional medical providers suggest that cancer diagnosis triggers intensified information seeking as patients confront decision gravity and complexity. However, this intensification takes the form of accumulating additional sources rather than replacing initial ones, indicating that patients employ triangulation strategies to build confidence and resolve uncertainty [39, 40].

### Trust and readiness barriers to AI-supported decision-making

The persistent underutilization of AI tools, even among younger, digitally engaged patients, raises questions about whether current AI applications adequately address patients’ need for trustworthy, personalized guidance during emotionally charged medical decisions [41, 42]. It suggests that technological innovation alone is insufficient; tools must align with patients’ decision-making goals, information-processing capacities, and trust-building requirements [43, 44]. The timing of data collection should also be considered when interpreting the low uptake of AI tools observed here. Data were collected between February 2025 and December 2025, relatively early in the public diffusion of consumer-facing generative AI. Adoption has since accelerated: recent US surveys indicate that nearly three in ten adults now use AI chatbots for health information or advice at least monthly, almost double the share reported in mid-2024 [46, 47]. As availability and familiarity increase, patients’ engagement with AI-based tools along the cancer-care journey may grow considerably, underscoring the need for repeated assessment of these behaviors over time.

### Decision-making dilemmas as a system-level challenge

The report of significant decision-making dilemmas by 86% of the patients reframes decision difficulty as a systemic issue rather than individual patient deficit. Treatment choice and provider selection emerged as dominant challenges, reflecting fundamental tensions in contemporary healthcare: increasingly complex treatment options requiring sophisticated risk–benefit assessments, fragmented delivery systems requiring patients to navigate provider networks, and limited time for deliberation despite high-stakes consequences. Patients’ descriptions of feeling “lost” and “paralyzed” suggest that current health-system structures—which emphasize informed consent and shared decision-making—may inadvertently shift the decisional burden onto patients without providing adequate scaffolding for deliberation. This aligns with criticisms of idealized shared decision-making models that assume that patients possess resources (time, health literacy, emotional capacity) to engage in extensive deliberation, when in reality many patients face significant constraints [45].

### Implications for practice and policy

Our findings suggest moving from universal protocols toward differentiated decision support that matches patients’ information-seeking styles and decision-making preferences. Brief screening at diagnosis could identify archetypes and trigger tailored interventions: algorithm-supported decision aids for Digital Natives; facilitated family meetings for Family-Centered patients; physician-guided deliberation with cultural sensitivity for Medical Professional-Focused patients. Healthcare systems must recognize that culturally responsive care extends beyond language translation to encompassing different epistemological frameworks pertaining to medical knowledge, decision authority, and information seeking.

### Generalizability across healthcare systems and cultural contexts

This study was conducted in Israel, and the specific configuration of archetypes and dilemmas likely reflects features of that context: universal health coverage with low financial barriers to care, a technologically advanced health system with high digital penetration, and a population with pronounced religious and cultural diversity. The pathways identified here may therefore manifest differently in other settings. In multi-payer systems such as the USA, dilemmas surrounding provider and facility selection may be intensified by insurance networks, coverage restrictions, and out-of-pocket costs, whereas in single-payer systems with strong gatekeeping such choices may be more constrained. In low- and middle-income countries, limited digital infrastructure, lower digital and health literacy, travel and cost barriers, and oncology workforce shortages may reduce the prevalence of digitally oriented archetypes and increase reliance on family networks and physician authority [3]; conversely, where access to specialists is scarce, informal and community sources may assume an even larger role. Family-centered decision-making is also likely to be more prominent in collectivist cultural contexts, in which diagnosis disclosure and treatment decisions are often mediated by relatives [11]. We would therefore expect the existence of heterogeneous information-seeking archetypes—and the inadequacy of one-size-fits-all engagement models—to generalize across settings, while the relative size of each archetype, the sources available to patients, and the dominant dilemmas will vary with healthcare financing, digital access, and cultural norms. Replication across health systems, including low- and middle-income countries and multi-payer models, is needed to test this expectation.

### Strengths and limitations

This study’s strengths include a comprehensive multisource assessment, a pre/post-comparison capturing temporal evolution, mixed-methods integration of quantitative patterns with qualitative experiences, and inclusion of participants representing diverse cancer types. The sample size provided adequate statistical power for archetype identification and allowed meaningful comparisons across patient subgroups.

Limitations should also be acknowledged. First, the cross-sectional design limits causal inference. Second, the study was conducted within the Israeli healthcare context, characterized by universal healthcare coverage and specific cultural and religious diversity, which may limit generalizability to other healthcare systems. Third, reliance on self-report measures may introduce recall and social desirability biases. Fourth, recruitment through online patient communities and Facebook groups may have introduced selection bias, potentially oversampling patients who are more digitally engaged, health-literate, or proactive in seeking information and support. In addition, convenience sampling may have further contributed to the overrepresentation of highly involved patients. Finally, the study focused on the early diagnostic period and therefore does not capture decision-making processes occurring during later stages of cancer care.

Future research should employ longitudinal designs, evaluate archetype-tailored interventions in randomized or pragmatic trials, expand to diverse healthcare systems and cultural contexts, and further investigate mechanisms underlying the underutilization of AI-based tools.

## Conclusion

Cancer patients exhibit substantial heterogeneity in information-seeking behaviors and decision-making approaches during the critical period from symptom onset to treatment initiation. Four distinct archetypes reflect fundamentally different pathways for navigating medical uncertainty, with religiosity emerging as a powerful cultural differentiator alongside demographic factors. The normative prevalence of decision challenges, combined with archetype diversity, demonstrates that true patient-centered cancer care must move beyond one-size-fits-all approaches toward differentiated support systems that respect varied cultural frameworks, information preferences, and decision-making values. To realize this vision, healthcare systems must recognize and accommodate diversity, rather than impose uniform models of patient engagement.

## Supplementary Information

Below is the link to the electronic supplementary material.

ESM 1 (DOCX 33.6 KB)

## Data Availability

The datasets generated and/or analyzed during the current study are not publicly available due to participant privacy considerations and the sensitive nature of the data, but are available from the corresponding author on reasonable request, subject to ethical approval and data-sharing requirements.
