# Supplementary material for: Beyond one-size-fits-all: mapping information-seeking and decision-making pathways in cancer care
Source: Support Care Cancer. 2026 Sep 22;34(10):1003. doi: 10.1007/s00520-026-11250-4 (PMC13597558; doi:10.1007/s00520-026-11250-4)
Supplement: Supplementary file 1 [file 520_2026_11250_MOESM1_ESM.docx]

**Online resources**

1. **Guide for the semi-structured interviews**

- Tell me a little about yourself, including past illnesses, family members, community, and children.
- Tell me more about how your cancer was discovered. Was it something ongoing or sudden?
- Who did you rely on during the diagnosis process? (clinical, family, social factors)
- Tell me about the symptoms you experienced.
- When did you first go to the doctor? Did the doctor suspect it was cancer?
- Tell me about any challenges or difficulties you faced during the diagnostic process.
- What tests or examinations did the doctor conduct or refer you to?
- Were you referred to a specialist? If so, which type and when?
- How did you choose your oncologist?
- At this stage, did you consult with friends or family, search online, or seek a second opinion?
- If you consulted multiple doctors, how did you decide who would treat you?
- Were there conflicting opinions (e.g., one doctor suggesting a specific drug and another recommending surgery or a different drug)? If so, how did you make your decision?
- What important non-medical information did you receive at this stage (e.g., from social networks, WhatsApp groups, Rabbi Firer, etc.)?
- Which treatment plan did you choose? Was it clear to you when you started?
- On which date did you begin treatment?

2. **Sociodemographic and clinical characteristics of the 37 patients who completed the pilot Patient Decision Agents Questionnaire**

| **Characteristic** | **Value** |
| --- | --- |
| **Number of patients** | 37 |
| **Gender, n (%)**  Male  Female  Missing values | 10 (27.0)  26 (70.3)  1 (2.7) |
| **Age range in years (mean, SD)** | 28–83 (61.4, 15) |
| **Family status, n (%)**  Married  In a relationship  Single  Widowed  Divorced | 25 (67.6)  6 (16.2)  1 (2.7)  2 (5.4)  3 (8.1) |
| **Religiosity, n (%)**  Secular  Traditional  Religious | 31 (83.8)  4 (10.8)  2 (5.4) |
| **Years of education range (mean, SD)** | 12–23 (16.5, 2.7) |
| **Average income, n (%)**  Far below average  Below average  Around average  Above average  Far above average | 2 (5.4)  9 (24.3)  9 (24.3)  14 (37.8)  1 (2.7) |
| **Employment status, n (%)**  Employed  Unemployed  Retiree/pensioner  Self-employed  Missing values | 9 (24.3)  3 (8.1)  20 (54.1)  3 (8.1)  2 (5.4) |
| **Additional private insurance, n (%)**  Yes  No  Missing values | 28 (75.7)  8 (21.6)  1 (2.7) |
| **Diagnosis year range (mean, SD)** | 1997–2024 (2015, 8) |
| **Cancer staging in diagnosis, n (%)**  Initial  Advanced  Missing values | 23 (62.2)  11 (29.7)  3 (8.1) |
| **Cancer types, n (%)**  Breast  Melanoma  Thyroid  Ovary  Non-Hodgkin lymphoma/brain lymphoma  Leiomyosarcoma/osteosarcoma  Salivary gland  Kidney  Myxoma  Prostate  Endometrium  Uterus – ovaries  Oligodendroglioma  Missing values | 18 (48.6)  2 (5.4)  2 (5.4)  2 (5.4)  2 (5.4)  2 (5.4)  1 (2.7)  1 (2.7)  1 (2.7)  1 (2.7)  1 (2.7)  1 (2.7)  1 (2.7)  2 (5.4) |

3. **Reliability analysis of the Patient Decision Agents Questionnaire**

| **Item** | **Mean** | **Cronbach's alpha if item deleted** |
| --- | --- | --- |
| **Patients’ sources of information before diagnosis:** | | |
| Family doctor | 1.90 | .871 |
| Medical professionals | 2.66 | .868 |
| Family members/partner | 3.00 | .862 |
| Friends/community | 2.38 | .865 |
| Religious/spiritual figure | 1.14 | .869 |
| Social media sources | 1.38 | .867 |
| Patient groups | 1.24 | .870 |
| Alternative/complementary medicine | 1.45 | .869 |
| Medical databases | 1.69 | .862 |
| General internet searches | 2.07 | .862 |
| GenAI tools | 1.00 | .871 |
| **Patients’ sources of information after diagnosis:** | | |
| Family doctor | 1.79 | .869 |
| Oncologist | 3.62 | .875 |
| Medical professionals | 2.55 | .869 |
| Family members/partner | 3.14 | .866 |
| Friends/community | 2.07 | .862 |
| Religious/spiritual figure | 1.28 | .871 |
| Social media sources | 1.38 | .868 |
| Patient groups | 1.24 | .870 |
| Alternative/complementary medicine | 1.55 | .869 |
| Medical databases | 1.86 | .863 |
| General internet searches | 2.45 | .866 |
| GenAI tools | 1.00 | .872 |
| **All questions** | **1.96** | **0.873** |

4. **Patient Decision Agents Questionnaire**

**Introductory Sociodemographic Information Questionnaire**

Below are general questions:

1. Gender: 1) Male 2) Female 3) Other, specify _______.
2. Your country of birth: 1. Israel 2. Other, specify ________________.
3. Year of birth: _______________.
4. Place of residence: Drop-down list with the option to start typing the location name.
5. Marital status: 1) Married 2) In a relationship 3) Single 4) Divorced 5) Separated 6) Widowed 7) Other, specify _____.
6. Religion: 1) Jewish 2) Muslim 3) Christian 4) Druze 5) Other, specify _____________________.
7. How would you define your religious affiliation? a) Secular b) Traditional c) Religious d) Ultra-Orthodox e) Other, specify _______.
8. The current average monthly wage (according to the Bureau of Statistics) is 12,379 NIS. Is your average monthly salary: 1) Much below average 2) Below average 3) Around average 4) Above average 5) Much above average.
9. Years of education: ____________________________.
10. Employment status: 1) Self-employed 2) Employed 3) Retired 4) Unemployed 5) Other, specify ____________________.
11. Health insurance provider: 1) Clalit 2) Maccabi 3) Meuhedet 4) Leumit 5) IDF 6) Other , specify____.
12. Supplemental insurance from your health insurance provider: 1) No 2) Yes, specify __________.
13. Private insurance: 1) No 2) Yes, specify ____________________.
14. On what date did your journey with cancer begin? ____________.
15. Diagnosed with cancer type ________ since ________.
16. When you were diagnosed with cancer, what stage was it?

a) Early stage

b) Advanced stage

c) Other _______.

**Part A – Start of the Treatment "Journey"**

Try to recall the start of your journey and answer the following questions:

1. Did you experience any early symptoms or signs?

a) Yes

b) No

c) Other__________.

2. What led you to seek medical evaluation?

a) Routine check-up

b) Other(, specify?) ____________.

3. Which medical professional did you approach after the routine check-up or when symptoms began regarding your health status?

a) Family doctor

b) Specialist doctor

c) Emergency room/hospital or urgent care/community medical center

d) Other(, specify?) ________.

4. What happened after your visit? (you can select more than one answer)

a) I was treated without a referral to another professional

b) I was referred for diagnostic tests in the community (e.g., CT or MRI)

c) I was referred for diagnostic tests at hospital outpatient clinics

d) I was referred to a specialist in the community or at the hospital

e) I was referred to the emergency room/hospitalization

f) I sought a private opinion

g) Other(, specify?) ______.

5. If you were referred to the emergency room (you can select more than one answer):

a) I underwent diagnostic tests and was released for further examination in the community

b) I underwent diagnostic tests and was hospitalized for further treatment

c) Other, specify ________

d) Not applicable.

6. What happened afterward? (you can select more than one answer):

a) I was sent for additional tests

b) I was referred for consultation with another specialist in the community or at the hospital

c) I returned to my family doctor with the results

d) I was referred to the emergency room/hospitalization

e) I was referred for cancer treatment (surgery/radiation/medication)

f) I sought another private opinion.

g) Other, specify _______________.

7. At this stage, did you receive a definitive cancer diagnosis (e.g., through imaging or biopsy results)?

a) Yes

b) No c)

Other, specify ________.

8. What was the next step after your cancer diagnosis? (you can select more than one answer):

a) I was referred to an oncologist at the hospital

b) I was referred to an oncologist in the community

c) I underwent additional tests

d) Other, specify _______________.

9. What was the next step after meeting with the oncologist? (you can select more than one answer):

a) I underwent additional tests at the hospital

b) I was referred for further consultation with another specialist at the hospital

c) We began discussing the treatment plan, and I was released

d) I started treatment at the hospital

e) Other, specify ________________.

**Part B – Factors Influencing Decision-Making**

When you searched for information during your treatment journey, which factors did you use? Please rate the answers from 1 to 5 (1 – Did not use at all, 5 – Used to a very high degree). Mark the appropriate number in each box.

1. Before you were diagnosed but when symptoms began or when examinations started, which factors did you use to seek information and make decisions?

| **Factors** | **Did not use at all** | **Used minimally** | **Used moderately** | **Used to a high degree** | **Used to a very high degree** |
| --- | --- | --- | --- | --- | --- |
| Family doctor |  |  |  |  |  |
| Other medical professionals |  |  |  |  |  |
| Family |  |  |  |  |  |
| Friends/community |  |  |  |  |  |
| Religious/spiritual figure |  |  |  |  |  |
| Social networks (Facebook, Instagram, etc.) |  |  |  |  |  |
| Patient support groups |  |  |  |  |  |
| Non-conventional medicine (Chinese medicine, homeopathy, naturopathy, etc.) |  |  |  |  |  |
| Medical databases |  |  |  |  |  |
| General internet search |  |  |  |  |  |
| AI tools |  |  |  |  |  |
| Other, specify _________ |  |  |  |  |  |

2. At what stage during diagnosis did you use the factor you rated highest?

a) Onset of symptoms

b) Family doctor/first doctor visit

c) Diagnostic tests

d) Oncologist appointment

e) Other ________.

3. After the cancer diagnosis and before starting initial treatment, which factors did you use to seek information and make decisions?

| **Factors** | **Did not use at all** | **Used minimally** | **Used moderately** | **Used to a high degree** | **Used to a very high degree** |
| --- | --- | --- | --- | --- | --- |
| Family doctor |  |  |  |  |  |
| Oncologist |  |  |  |  |  |
| Other medical professionals |  |  |  |  |  |
| Family |  |  |  |  |  |
| Friends/community |  |  |  |  |  |
| Religious/spiritual figure |  |  |  |  |  |
| Social networks (Facebook, Instagram, etc.) |  |  |  |  |  |
| Patient support groups |  |  |  |  |  |
| Non-conventional medicine (Chinese medicine, homeopathy, naturopathy, etc.) |  |  |  |  |  |
| Medical databases |  |  |  |  |  |
| General internet search |  |  |  |  |  |
| AI tools |  |  |  |  |  |
| Other, specify _________ |  |  |  |  |  |

4. During the time from diagnosis to starting treatment, when did you use the factor you rated highest?

a) Oncologist meeting

b) Additional consultations

c) Additional diagnostic tests

d) Initial treatment planning

e) Other, specify ________.

**Part C – Decision Points and Decision-Making**

1. How did you choose your treating oncologist? (you can select more than one answer):

a) The health insurance provided a list, and I chose from it

b) I was hospitalized and did not choose the oncologist

c) My family doctor recommended a specific doctor, and I followed the recommendation

d) I received a recommendation from a family member

e) I received a recommendation from a friend

f) I received a recommendation from a social media group

g) I consulted Rabbi Firer or another advisor who gave me a recommendation

h) I searched online and found a suitable doctor

i) Proximity to my home

j) I used AI search engines

k) I visited several oncologists and chose the one I connected with personally

l) Other, specify ________.

2. If you had multiple opinions and they differed, how did you decide which opinion to follow?

a) I did not seek a second opinion

b) The opinions were similar

c) I consulted with family members

d) I consulted with friends

e) I read online and made the decision myself

f) I read medical articles

g) I consulted a religious/spiritual figure

h) Other, specify ________.

3. Who managed your treatment journey from diagnosis to the start of initial treatment?

a) I managed it

b) A family member

c) Friends/relatives

d) Paid medical management services

e) My family doctor

f) Other, please specify ____________.

4. If you could identify the most influential factor in your decision-making during your journey, what would it be?

a) My own personality/only me

b) My family doctor

c) My oncologist

d) A family member

e) Patient support groups/organizations

f) Information on social networks

g) Information from AI tools

h) Other, please specify ________________.

5. Can you recall a dilemma that arose during the initial phase of your journey and how you resolved it? (open-ended) __________________________________________________.
